# Knowledge-Graph-Driven Fault Diagnosis Methods for Intelligent Production Lines

**DOI:** 10.3390/s25133912

**Published:** 2025-06-23

**Authors:** Yanjun Chen, Min Zhou, Meizhou Zhang, Meng Zha

**Affiliations:** 1 Key Laboratory of Metallurgical Equipment and Control Technology, Ministry of Education, Wuhan University of Science and Technology, Wuhan 430081, China; yanjunchen521@wust.edu.cn (Y.C.); zhangmeizhou@wust.edu.cn (M.Z.); zhameng527479113@wust.edu.cn (M.Z.); 2 Hubei Key Laboratory of Mechanical Transmission and Manufacturing Engineering, Wuhan University of Science and Technology, Wuhan 430081, China; 3 Institute of Precision Manufacturing, Wuhan University of Science and Technology, Wuhan 430081, China

**Keywords:** knowledge graph, intelligent production line, fault diagnosis, entity recognition, BiLSTM (bidirectional long short-term memory network)

## Abstract

In order to enhance the management and application of fault knowledge within intelligent production lines, thereby increasing the efficiency of fault diagnosis and ensuring the stable and reliable operation of these systems, we propose a fault diagnosis methodology that leverages knowledge graphs. First, we designed an ontology model for fault knowledge by integrating textual features from various components of the production line with expert insights. Second, we employed the ALBERT–BiLSTM–Attention–CRF model to achieve named entity and relationship recognition for faults in intelligent production lines. The introduction of the ALBERT model resulted in a 7.3% improvement in the *F*_1_ score compared to the BiLSTM–CRF model. Additionally, incorporating the attention mechanism in relationship extraction led to a 7.37% increase in the *F*_1_ score. Finally, we utilized the Neo4j graph database to facilitate the storage and visualization of fault knowledge, validating the effectiveness of our proposed method through a case study on fault diagnosis in CNC machining centers. The research findings indicate that this method excels in recognizing textual entities and relationships related to faults in intelligent production lines, effectively leveraging prior knowledge of faults across various components and elucidating their causes. This approach provides maintenance personnel with an intuitive tool for fault diagnosis and decision support, thereby enhancing diagnostic accuracy and efficiency.

## 1. Introduction

The intelligent production line system, which serves as the foundation of smart manufacturing, comprises multiple interconnected production units. The stable and efficient operation of this system is essential for maintaining production efficiency and ensuring product quality. However, prolonged operation under high loads and in complex environments often leads to frequent failures, which pose significant risks to the system’s safety, economic viability, and reliability [1].

Failures within production lines can be classified into two primary categories: mechanical system failures and electrical system failures. Diagnosing these failures is a complex and meticulous endeavor, as they are inter-related and require comprehensive analysis to identify root causes [2]. Traditional fault diagnosis and health management approaches predominantly rely on the assessment of relevant signals or the extraction of features, such as vibration acceleration signals, acoustic signals, and thermal signals. When mechanical and electrical components of the production line fail, error messages and alarm codes are typically generated; however, this information is often limited to basic system or electrical data and is frequently presented in unstructured text formats [3]. This limitation complicates the integration and application of dispersed knowledge, resulting in an over-reliance on human expertise. Such dependence not only diminishes the effective utilization of knowledge but also hinders the sharing of expertise among maintenance personnel, thereby complicating comprehensive diagnosis. Furthermore, this situation exacerbates the issue of information silos, significantly impairing the accuracy and efficiency of fault diagnosis and leading to a notable decline in diagnostic precision. Consequently, these challenges result in low diagnostic efficiency and slow advancements in diagnostic capabilities, making it difficult to address the pressing demands of contemporary production environments. Therefore, in light of the rapid evolution of industrial technology, it is imperative to explore methodologies that ensure the safe and stable operation of electromechanical equipment within intelligent production lines. The advancement of effective and accurate fault diagnosis technologies for intelligent production line systems is essential to meet the varied demands associated with equipment status analysis, fault prediction, risk assessment, and the optimization of maintenance practices.

In contrast to the previously mentioned methods, knowledge graphs [4] represent an advanced form of graphical knowledge representation derived from directed graphs. This technical approach employs graph models to depict knowledge and illustrate the relationships between entities and concepts. Knowledge graphs facilitate the identification, discovery, and inference of complex relationships among entities and concepts derived from data, thereby enabling the extraction of relevant information [5]. By leveraging knowledge graph technology, it becomes possible to analyze the challenges associated with fault diagnosis in intelligent production lines from multiple perspectives, ultimately leading to effective problem resolution.

The contemporary concept of knowledge graphs was introduced by Google in 2012. Fundamentally, it represents a semantic network of entity relationships and is currently associated with large-scale databases [6]. Knowledge graphs are widely utilized in search engines, question-answering systems, decision-making processes, and artificial intelligence reasoning. As a structured method of knowledge representation, knowledge graphs (KGs) organize complex information in a graphical format, thereby enhancing the ability of computers to store, retrieve, and infer knowledge more effectively.

Knowledge graphs consist of nodes and directed edges, where each node represents an entity and can be expressed as a set of triples (*E*_1_, *R*, and *E*_2_) [7]. In this representation, *E*_1_ denotes the head entity, *E*_2_ signifies the tail entity (the nodes within the knowledge graph), and R represents the relationship between the two entities, corresponding to the edges in the graph. The set of entities is denoted as *E* = {*e*_1_, *e*_2_, …, *e_i_*, …, *e_n_*}, where eᵢ represents the *i-th* entity. The set of relationships is denoted as *R* = {*r*_1_, *r*_2_, …, *r_j_*, …, *r_m_*}, where *r_j_* represents the *j-th* relationship. The construction of knowledge graphs incorporates various key technologies, including knowledge modeling, entity extraction (e.g., named entity recognition, NER), relationship extraction, knowledge fusion, graph storage, and relationship reasoning.

Given its advantages of clear semantics, rich information, and structured clarity, knowledge graph technology offers effective technical means for the extraction, representation, storage, and reasoning of fault knowledge, thereby demonstrating its unique value [8]. This technology integrates knowledge resources from various fields to construct a comprehensive and systematic knowledge system that encompasses not only the structural composition, operational principles, and performance indicators of production units but also the categories, characteristics, causes, and diagnostic strategies of faults [9].

To ensure the long-term safe and stable operation of electromechanical equipment on intelligent production lines, it is essential to implement regular maintenance and upkeep measures, as well as to maintain detailed records of equipment status and maintenance history [10]. These log records contain valuable data that can assist in status analysis, fault prediction, risk assessment, and maintenance optimization. However, the textual nature of log records presents challenges, including a lack of standardized structure, diverse recording methods, and stylistic variations. The diversity and complexity of textual data pose significant obstacles to the entity extraction task in knowledge graph construction. In this context, the effectiveness and accuracy of entity extraction directly influence the quantity and quality of data available for subsequent knowledge modeling. Therefore, the importance of researching entity extraction methods for knowledge graph construction is evident.

Effectively harnessing a substantial amount of unstructured expert text data to assess and predict the operational status of intelligent production lines is critical for smart manufacturing. By identifying entities from operational, running, and maintenance texts related to intelligent production lines; extracting relationships between these entities to form triples; and subsequently importing these triples into a graph database, it is possible to create a knowledge graph of faults in intelligent production lines. This process facilitates the transition from unstructured text data to structured data. In this knowledge graph, nodes represent various entities within the production line domain, including equipment, fault types, causes, and solutions, while edges illustrate the relationships between these entities, such as inclusion and correlation [11].

The intelligent production line fault knowledge graph not only presents high-quality knowledge information but also facilitates convenient querying and intuitive visualization of equipment fault knowledge. Moreover, it serves as a prerequisite for intelligent semantic understanding in the field of production line fault diagnosis, providing theoretical data support for subsequent knowledge reasoning related to equipment faults. The fault diagnosis technology that integrates knowledge graphs can accurately characterize the faults of various components within the intelligent production line and perform logical reasoning. Firstly, it aids maintenance personnel in the precise identification of system faults and their underlying causes, which facilitates more informed decision making, promotes safety awareness, effectively reduces economic losses associated with faults, and ensures the uninterrupted and safe operation of production processes. Secondly, it diminishes the reliance on specialized knowledge, compensating for the inexperience of staff by accurately diagnosing on-site equipment faults through the analysis of historical fault records and their potential causes. This capability significantly lowers the incidence of misjudgments and missed diagnoses, effectively addressing a range of accidents and faults, and further enhancing the safety and stability of the intelligent production line, thereby ensuring the continuity and efficiency of the production process.

However, challenges such as ambiguity, synonyms, entity nesting, and uneven data distribution hinder Chinese entity recognition and relationship extraction in the context of intelligent production line fault diagnosis [12]. Despite advancements in technology and the promotion of the industrial internet, which have enabled real-time monitoring and precise predictive analysis of equipment operating status, the field remains constrained by the lack of publicly available data and the complexities involved in effectively extracting fault diagnosis knowledge.

To address these challenges, this paper proposes a method for constructing a knowledge graph aimed at intelligent production line fault diagnosis, utilizing the ALBERT model. Initially, we collect the publicly available literature in the field of intelligent production lines and employ semi-structured corpora for sample expansion to develop a training dataset specifically designed for fault diagnosis in intelligent production lines. Subsequently, we implement the ALBERT–BiLSTM–CRF deep learning algorithm, which has demonstrated superior performance in named entity recognition tasks. Through the extraction of operational and maintenance entities from the literature pertaining to production lines and accident reports, we assess the efficacy of our approach by juxtaposing our findings with those derived from alternative deep learning algorithms. This comparison serves to substantiate the effectiveness and benefits of our method in improving the accuracy and recall rates of entity recognition. In the task of extracting relationships among production line operation and maintenance entities, we utilize the ALBERT–BiLSTM–Attention deep learning algorithm, which integrates ALBERT and attention mechanisms and exhibits significant advantages in processing time-series data and extracting key features. Compared to other deep learning algorithms, this approach demonstrates superior performance in text processing tasks within the realm of intelligent production line fault diagnosis. Finally, we visualize the knowledge graph using the Neo4j graph database and validate it through application cases, achieving an auxiliary decision-making function based on the intelligent production line operation and maintenance knowledge graph.

**Figure 1:**
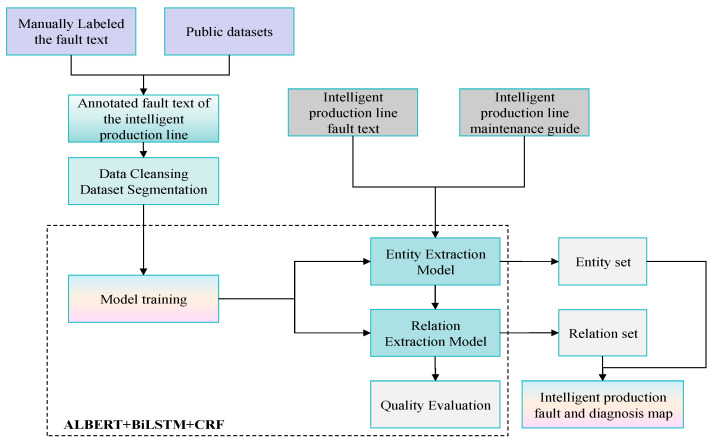
Overall architecture diagram.

Figure 1 illustrates the comprehensive architecture diagram. Initially, labeled texts related to faults in intelligent production lines are sourced from public datasets and manually annotated materials. Subsequently, preprocessing operations are conducted on these texts, which include the removal of special symbols and verification of the accuracy of the annotation format. The entire dataset is then partitioned into three segments for training, validation, and testing purposes. Following this, the ALBERT + BiLSTM + CRF model is employed for model training. Through meticulous evaluation and optimization of the algorithm, the system can process a substantial volume of texts related to intelligent production line faults or maintenance guidance data. These texts are then input into the proposed algorithm to yield precise prediction outcomes and derive structured entity sets and relationship sets. After eliminating duplicates, these data are systematically organized and consolidated to form triples. Ultimately, a knowledge graph database dedicated to diagnosing faults in intelligent production lines is established.

The remainder of this paper is organized as follows: The second section provides an overview of the exploration and research achievements related to knowledge graphs in the field of fault diagnosis. The third section discusses the research methods, primarily introducing the overall framework of the model and detailing each key module. The fourth section elaborates on the sources and construction process of the model dataset, the selection of evaluation metrics, and parameter settings, while conducting an in-depth comparison and analysis of its performance. The fifth section presents the constructed knowledge graph and validates it through application cases. The sixth section discusses the purpose and significance of the research, describes its limitations, and outlines future research directions. The seventh section summarizes the paper, while the eighth section discloses the status of related Chinese patents.

## 2. Related Work

The academic and industrial sectors are increasingly exploring knowledge graphs, with a growing number of researchers applying these frameworks to fault diagnosis. In the context of diagnosing electrical equipment faults, scholars are integrating operational data, fault case studies, and expert insights to develop fault diagnosis rules and reasoning models [13]. This integration enhances the intelligence of existing systems and fosters innovation within the industry. Key entities relevant to equipment fault diagnosis include equipment names, fault locations, fault phenomena, fault causes, and fault remediation methods. In contrast to entity recognition in other domains such as finance, healthcare, and agriculture, the entities associated with equipment fault diagnosis tend to be more extensive and filled with specialized terminology [14]. Furthermore, the limited availability of public datasets and the complexity of nested entity types exacerbate the challenges associated with named entity recognition in this field.

In their research, Wang et al. [15] extracted semi-structured and unstructured fault knowledge through an in-depth analysis of CNC machine tool fault cases, maintenance manuals, and field logs. They successfully implemented structured applications for CNC machine tool fault diagnosis by employing a variety of diagnostic techniques, including action inspection, state analysis, programming inspection, instrument inspection, and system self-diagnosis. Cai et al. [16] introduced a multi-level knowledge graph construction methodology to enhance fault diagnosis. They further developed a system state detection approach based on multi-level knowledge graphs and Bayesian theory, utilizing relationship path-based reasoning to identify fault sources. By leveraging the inter-relationships among the structures of rotating machinery to deduce fault causes and employing knowledge graphs as a reasoning knowledge base for machine learning, they significantly improved the accuracy of fault diagnosis.

Deng et al. [17] investigated strategies for constructing knowledge graphs aimed at fault diagnosis in robotic transmission systems. They utilized a Bidirectional Long Short-Term Memory (BiLSTM) network to capture contextual features in fault-related texts, a self-attention mechanism to accurately extract interdependencies among characters in multi-dimensional subspaces, and a Conditional Random Field (CRF) for effective recognition of key entities, thereby facilitating autonomous fault diagnosis. Liu et al. [18] created a knowledge graph for electrical equipment faults based on operational and maintenance records, illustrating the correlation between faulty equipment and its components. Named entity recognition is essential for constructing the knowledge graph for electrical equipment fault diagnosis. Zuo et al. [19] proposed an X-ray-based multi-expert detection method for the automatic assessment of welding defects in intelligent pipeline systems, thereby enhancing the efficiency and accuracy of pipeline welding fault diagnosis. Chen et al. [20] introduced a benchmark dataset and baseline model that employs sequence labeling (SL) and named entity recognition (NER) techniques to extract fault diagnosis knowledge from compressor maintenance logs.

Gong et al. [21] introduced a BiLSTM and CRF model based on BERT, which combines a BERT–BiLSTM with a CCA–CRF model to identify entities related to high-voltage isolating switches and thermal faults in electrical grid fault texts.

Han, T. et al. [22] significantly enhanced the accuracy of constructing aeroengine fault knowledge graphs by employing a Lattice Transformer–CRF entity extraction method that integrates word sequence information. This advancement facilitates the application of knowledge graphs in fault diagnosis. The successful implementation of automated and intelligent fault diagnosis has been achieved by leveraging knowledge graphs in conjunction with sophisticated fault diagnosis algorithms.

Meng et al. [23] effectively extracted interdependencies among relevant entities and relationships within the aircraft health management system using a joint recognition model based on sequences and TreeBiLSTM. This approach is significant for advancing research in intelligent health management systems for aircraft. Yang et al. [24] integrated BERT, BiLSTM, and CRF for the text mining of fault reports related to railway operational equipment and for extracting pertinent entities.

In the field of electrical equipment fault diagnosis, Meng et al. [25] employed a BERT–BiLSTM–CRF model that integrates deep learning techniques to identify and extract electrical equipment entities from preprocessed Chinese technical literature. This approach demonstrated significant improvements in the accuracy of entity extraction. Subsequently, dependency analysis and relationship classification techniques were utilized to further extract deep semantic relationships among the entities. The resulting knowledge is stored in a Neo4j database in the form of triples and is visualized graphically.

Shu, X. et al. [26] focused on fault entity relation extraction within the mine hoist system, successfully developing a maintenance support and fault diagnosis decision-making system that employs an ontology model. Cao, X.G. et al. [27] devised a top–down knowledge graph construction framework, systematically transforming fault maintenance data collection into the creation of a static, fully connected fault knowledge graph for coal mine electromechanical equipment. Utilizing a customized methodology and system architecture, the researchers successfully facilitated the seamless integration of raw data acquisition with structured knowledge representation, specifically tailored for fault diagnosis and maintenance applications in mining machinery.

Chen, Q. et al. [28] utilized the TextCNN model to extract critical information through Convolutional Neural Networks (CNNs). By employing multi-scale convolution kernels, the model effectively captures intricate local correlations within textual sequences, thereby significantly improving classification accuracy for power grid fault documentation. This methodology has been validated across various domains, including telecommunication equipment fault categorization and substation diagnostic systems. While TextCNN demonstrates a modest 1.9% accuracy improvement over BiLSTM–CRF models in power grid fault diagnosis scenarios, its exceptional capability in local feature extraction remains a vital asset in power system analytics.

Chen, H.T. et al. [29] employed the BiLSTM–CRF model to extract entities from fault descriptions in high-speed railway signaling systems, effectively demonstrating its practical applicability. However, the study overlooked a comparative analysis with alternative models, which is a critical step for comprehensively evaluating the performance and robustness of the BiLSTM–CRF model.

Research on fault diagnosis utilizing knowledge graphs has advanced significantly across various sectors, including power systems, transportation, and aerospace. This progress has markedly improved the efficiency of fault knowledge utilization and enhanced the accuracy of fault diagnosis. However, there remains a relative paucity of research focused on constructing knowledge graphs specifically tailored to the characteristics of fault text data in intelligent production lines.

This study focuses on multi-dimensional fault text data from intelligent production lines, systematically organizing and summarizing knowledge related to faults in these systems. It successfully constructs a knowledge graph system for intelligent production line faults using deep learning techniques. By extracting fault entities and their associated relationships, the study transforms unstructured data into structured formats for storage and management, utilizing the Neo4j database for visualization and in-depth analysis. This approach provides robust data support for system maintenance, assisting maintenance personnel in enhancing the efficiency and accuracy of fault diagnosis and analysis. The incorporation of knowledge graph-based fault diagnosis technology significantly mitigates the technical challenges associated with fault diagnosis in intelligent production line systems. This advancement enhances operational efficiency and safety while simultaneously augmenting the digital and intelligent capabilities of production lines. The key contributions of this paper are outlined as follows:

1. The collection and organization of fault diagnosis texts from the intelligent production line equipment fault database enabled the creation of a specialized Chinese corpus for this field. Additionally, samples from semi-structured corpora were expanded to develop a training dataset specifically designed for intelligent production line fault diagnosis. The text preprocessing tasks were completed through data cleaning, sentence segmentation, and meticulous annotation of entities, including equipment names, fault locations, fault phenomena, fault causes, and solutions. A novel method for constructing a knowledge graph for intelligent production line fault diagnosis based on ALBERT was proposed.
2. The ALBERT–BiLSTM–CRF deep learning algorithm was employed for named entity recognition, demonstrating exceptional performance in this domain. The efficacy and benefits of this method were substantiated by extracting operational entities from the relevant literature and accident reports pertaining to production lines, followed by a comparative analysis of its performance against other deep learning algorithms. This approach significantly enhanced the accuracy and recall rate of entity recognition in intelligent production line fault diagnosis. The model achieved precision, recall, and *F*_1_ scores of 92.57%, 94.35%, and 93.45%, respectively.
3. In the task of extracting relationships among operational entities in production lines, the ALBERT–BiLSTM–Attention deep learning algorithm was utilized. This algorithm combines ALBERT with attention mechanisms, emphasizing its strengths in processing time-series data and extracting essential features. It demonstrated superior performance in text processing tasks related to intelligent production line fault diagnosis when compared to other deep learning algorithms.
4. A specialized knowledge graph framework has been developed to diagnose faults in intelligent production line equipment, effectively addressing the challenges of knowledge storage and visualization in this domain. Various application cases have validated the auxiliary decision-making function based on the operational knowledge graph of the intelligent production line. This work also serves as a reference for the application of knowledge graphs in other engineering fields.

The future directions for enhancing fault diagnosis methods in intelligent production lines include the following aspects:

1. Model Structure Optimization. The current ALBERT–BiLSTM–Attention–CRF model demonstrates strong performance in fault diagnosis; however, there is considerable potential for optimization. Future research could explore the use of lighter pre-trained models to replace ALBERT, which would reduce the number of parameters and improve the inference speed. Additionally, the BiLSTM architecture could be enhanced by incorporating Gated Recurrent Units (GRUs) to replace some LSTM units, thereby decreasing model complexity. Furthermore, implementing a multi-task learning framework could be beneficial, enabling the model to simultaneously learn tasks such as fault diagnosis and equipment health status prediction while sharing underlying features to improve diagnostic accuracy.
2. Integration of Domain Knowledge and Dynamic Updating of Knowledge Graphs. Knowledge graphs play a crucial role in fault diagnosis, and their capacity for dynamic updates is essential. In the future, it may be beneficial to incorporate more expert knowledge from the field, such as physical models of equipment and specifications for process flows, to enhance the entities and relationships within the knowledge graph. Natural language processing technologies could be employed to analyze technical documents and maintenance records in real time, automatically extracting new information for integration into the graph. Furthermore, advanced graph-updating algorithms could be developed to automatically adjust knowledge weights based on fault diagnosis outcomes, reinforcing knowledge related to frequently occurring faults while diminishing the relevance of outdated information. This approach would ensure that the knowledge graph accurately reflects the current state of the production line.
3. Exploration of Efficient Computing Methods. To minimize the computational costs of models, distributed computing architectures can be employed by deploying various modules of the model across multiple servers for parallel processing. This approach reduces both training and inference times. Additionally, hardware acceleration technologies, such as GPUs and FPGAs, can be utilized to optimize computational processes based on the model’s operational characteristics, thereby enhancing overall efficiency.
4. Enhancing System Performance through the Integration of IoT and Big Data Technologies. The Internet of Things (IoT) gathers real-time operational data from devices, while big data analytics technologies process vast amounts of historical fault information to reveal hidden fault patterns and their correlations. This integration enables the development of knowledge graphs and the training of predictive models, providing more comprehensive and timely data support for fault diagnosis.

In summary, by optimizing model structures, deeply integrating domain knowledge, exploring efficient computing methods, and closely collaborating with other advanced technologies, intelligent production line fault diagnosis systems are anticipated to achieve improved accuracy, efficiency, and intelligence in fault diagnosis in the future.

## 3. Research Methodology

### 3.1. Construction Framework of Intelligent Production Line Knowledge Graph

In the construction of a knowledge graph, the concept of “graph” diverges from traditional representations. It serves as a structured representation, similar to a chemical molecular formula, illustrating the intricate relationships among data. The graph’s database primarily derives from textual data, with additional sources enhancing its descriptive accuracy. By integrating rich semantics, logical implications, and rules, it constructs a semantic network that can accurately depict complex relationships. The network employs triples, which can encompass and express various types of entities and their inter-relations [30]. This approach provides a structured and visual method for representing knowledge in nonlinear contexts, thereby improving the efficiency of human knowledge classification, prediction, and logical reasoning. This is evidenced by the application of nonlinear knowledge-based classification in neural output-feedback control.

**Figure 2:**
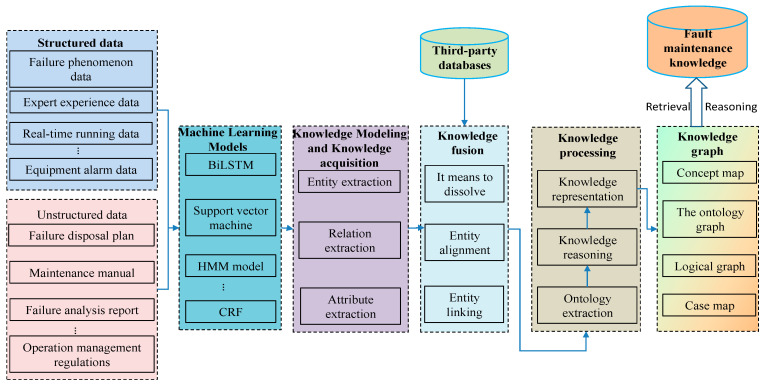
Fault knowledge map construction framework of intelligent production line.

The construction of a knowledge graph necessitates careful consideration of the intricate processes involving domain-specific contexts, semantic structures, and operational requirements. The methodology employed may encompass a top–down approach, a bottom–up strategy, or a seamless integration of both [31]. In developing a fault knowledge graph for an intelligent production line, this paper adopts a combined top–down and bottom–up strategy, as illustrated in Figure 2. The objective is to achieve high accuracy and a comprehensive representation of fault knowledge in intelligent production lines by leveraging the strengths of both methods. This strategy reflects the construction method utilized for equipment manufacturing fault knowledge graphs, ensuring systematic integrity while enhancing flexibility and adaptability in practical applications.

### 3.2. Intelligent Production Line Fault Diagnosis Ontology Construction

In the construction framework of the fault knowledge graph for intelligent production lines, fault knowledge ontology plays a crucial role by providing a comprehensive set of standardized definitions for concepts and attributes. This ensures the systematic management of these elements by clearly delineating concepts, entities, and attributes [32]. The process begins with the refinement of top-level concepts, followed by the definition of entity concepts, associations, and attributes to establish a set of ontology rules that create a standardized framework for extracting and integrating entity knowledge. The schema layer of the knowledge graph is governed by the ontology library, where the concepts and relationships defined by the ontology form the concept nodes and associations, serving as the theoretical and methodological foundation for constructing the knowledge graph.

**Figure 3:**
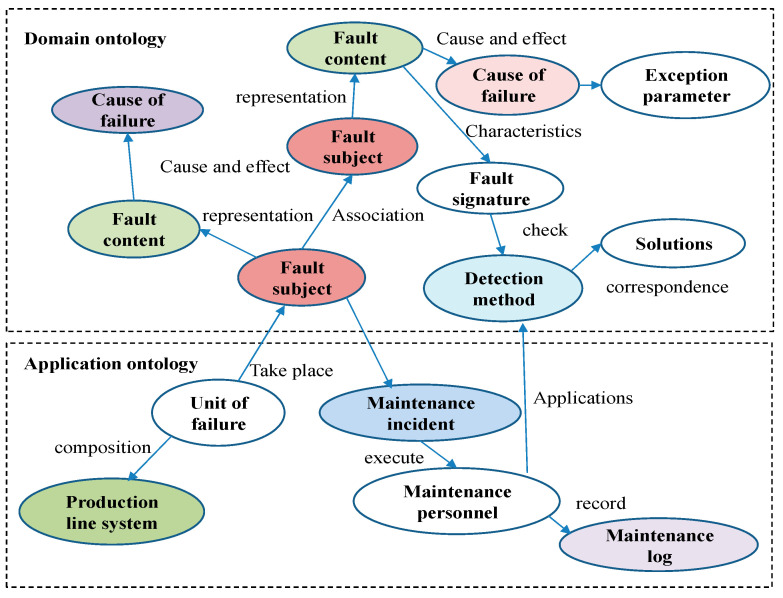
Part of the ontology association diagram.

In the field of intelligent production lines, we have utilized expert knowledge and the Protégé tool to develop a comprehensive knowledge graph for fault diagnosis. This knowledge graph includes various entity types, such as fault subjects, contents, causes, detection methods, and maintenance solutions, each accompanied by clearly defined relationship types and definitions for both head and tail entities. These definitions enrich the content of the knowledge graph and establish a robust semantic foundation for effective fault diagnosis. A partial representation of the ontology association relations is illustrated in Figure 3.

Domain ontology construction focuses on consolidating and refining knowledge related to faults, aiming to uncover the intricate interconnections among fault entities. It involves a systematic categorization of fault data and a precise depiction of the various relationships among these entities. In contrast, application ontology emphasizes transforming fault information into practical outcomes in real-world scenarios, striving to provide robust solutions. Building upon this solid foundation, the application ontology inherits the core characteristics of the domain ontology. While rigorously defining associative relationships, it meticulously delineates domains and specifies value ranges for both head and tail entities, thereby ensuring the logical consistency of these relationships. For instance, when addressing the “association” relationship, it is explicitly stated that both the head entity and tail entity must be “fault subjects”, ensuring the accuracy and credibility of the relationship within the knowledge graph.

### 3.3. Overall Structure of ALBERT–BiLSTM–Attention–CRF Model

**Figure 4:**
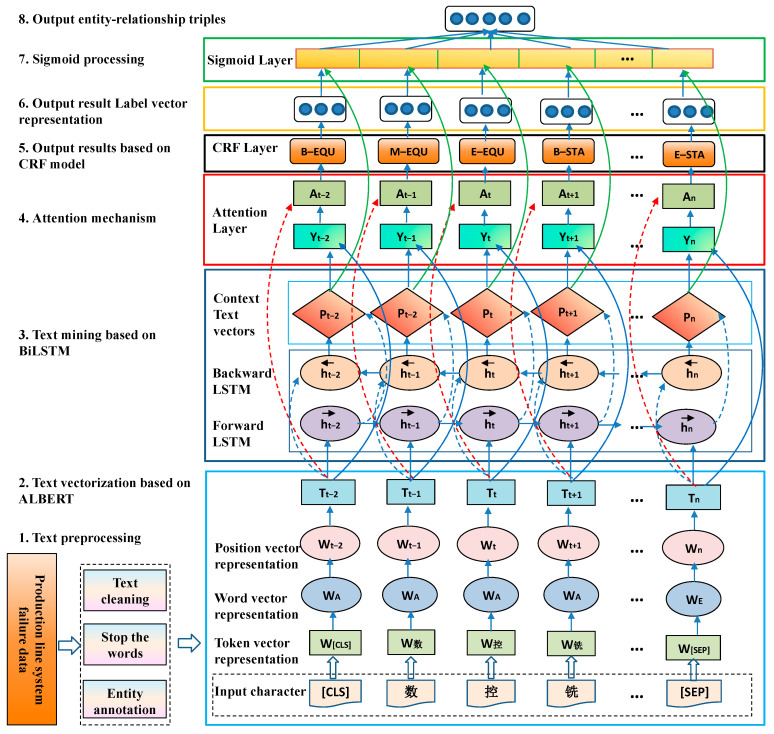
Fault knowledge extraction model of intelligent production line based on ALBERT–BiLSTM–Attention–CRF.

This study presents an advanced fault diagnosis model specifically designed for intelligent production lines. It integrates ALBERT, BiLSTM, attention mechanisms, and Conditional Random Fields (CRFs) to enhance its knowledge extraction capabilities. The model comprises eight essential components that encompass the entire workflow, from initial text preprocessing to the final stage of knowledge extraction, as illustrated in Figure 4. The text in Figure 4, “数 控 铣” means “CNC milling machine”.

1. Text Preprocessing: Preprocessing operations, including data cleaning and stop word removal, are conducted on the fault text data obtained from the intelligent production line system. These steps improve the quality of the data and prepare them for feature extraction.
2. Text Vectorization Using ALBERT: The ALBERT model vectorizes fault text from the intelligent production line system at the character level, converting it into high-quality word vectors that serve as the basis for subsequent model input.
3. Text Mining Based on BiLSTM: The BiLSTM model for text mining effectively captures contextual features in sequential data, generating initial results for named entity recognition that are subsequently fed into the attention layer.
4. Attention Mechanism: In the attention mechanism, the original word vector is combined with the text vector transformed by the BiLSTM layer. This process emphasizes the features that are most pertinent to the entity recognition task through weighted processing.
5. CRF Layer Classification Learning: The weighted vectors from the attention layer are input into the Conditional Random Field (CRF) layer for sequence classification, which enhances the accuracy of entity boundary recognition.
6. Entity Class Prediction: The incorporation of a Conditional Random Field (CRF) layer in sequence labeling significantly enhances the accuracy of entity recognition by taking into account the transition probabilities between labels. This approach establishes a robust foundation for precise entity classification.

7. Relation Entity Recognition: The entity category and context feature vectors are combined and subsequently processed by a sigmoid layer to identify and output relational entities.
8. Entity Relation Triples Generation: The model generates entity–relation triples after processing, which provide structured knowledge and information for constructing the knowledge graph.

By synergistically integrating its modules, the model effectively translates raw text into structured knowledge, offering a robust solution for knowledge extraction in the domain of intelligent production line fault diagnosis, as demonstrated by numerous real-world applications.

### 3.4. ALBERT–BiLSTM–Attention–CRF Knowledge Extraction Model Construction

As one of the fundamental technologies in the field of natural language processing, knowledge extraction aims to extract essential information from unstructured text data During the construction of a knowledge graph, knowledge extraction technology primarily focuses on identifying specific entities, attributes, and the intricate relationships they represent within the text. This study introduces a knowledge extraction model named ALBERT–BiLSTM–Attention–CRF, which is specifically designed for fault diagnosis in intelligent products.

The pre-trained ALBERT model refines word embeddings, enhancing character-level accuracy and establishing a foundation for knowledge extraction. By incorporating a Bidirectional Long Short-Term Memory (BiLSTM) network, which is renowned for its ability to capture contextual information, the model further improves semantic understanding among entities. Additionally, the integration of the attention mechanism significantly enhances the model’s capacity to identify critical information, thereby increasing the precision of recognizing key entities and attributes within the text. The incorporation of dropout within the Conditional Random Field (CRF) framework has markedly improved the model’s generalization capabilities, enhancing its adaptability and robustness when handling new data. The ALBERT–BiLSTM–CRF model demonstrates superior capabilities in concurrently executing entity recognition and relation extraction tasks. Furthermore, it proficiently extracts critical knowledge pertinent to the field of intelligent production line fault diagnosis, thereby offering significant assistance in fault prediction and diagnosis. Each module of the model is analyzed and discussed in detail below.

#### 3.4.1. ALBERT Model

The initial phase of entity relationship extraction involves converting textual data into embedded representations. The accuracy of the semantic features within these embedded representations is crucial for minimizing error propagation in subsequent processing stages. To enhance precision and address the challenges posed by the substantial size of the BERT [33] pre-trained model, this research has chosen the ALBERT model. This model effectively reduces the number of parameters while maintaining performance levels comparable to those of BERT by employing techniques such as parameter sharing and low-rank decomposition.

In 2020, Z. Lan et al. [34] introduced the ALBERT model, a lightweight, self-supervised learning pre-training optimization framework that utilizes a Transformer architecture similar to BERT while incorporating several enhancements. These improvements primarily result in a significant reduction in the number of parameters and innovations in the Next Sentence Prediction task. Specifically, the model enhances its understanding of the logical relationships between sentences by replacing the Next Sentence Prediction task with the Sentence Order Prediction (SOP) task.

Moreover, ALBERT (A Lite BERT) optimizes the BERT architecture by refining its network structure [35]. It employs matrix decomposition techniques to partition the word embedding parameters into two smaller matrices, significantly reducing the model’s parameter count. Additionally, ALBERT incorporates a cross-layer parameter sharing mechanism, which further decreases the parameter count by allowing parameters to be shared across different layers. Through these lightweight techniques, the ALBERT model markedly enhances training efficiency while maintaining performance, demonstrating superior results in major benchmark evaluations and thereby increasing the model’s robustness [36].

In ALBERT, the decomposed embedding parameterization is employed to reduce the number of parameters by factorizing the embedding parameters into two smaller matrices. The vocabulary size *V* words no longer directly correspond to the one-hot vector in the hidden space *H*; instead, words are first mapped to a low-dimensional embedding space *E* and subsequently to the hidden space. This process reduces the parameter count from *O*(*V* × *H*) to *O*(*V* × *E* + *E* × *H*). When the dimension of the hidden space *H* is significantly larger than that of the low-dimensional embedding space *E*, the effect of parameter reduction becomes pronounced. Furthermore, ALBERT implements parameter sharing within the hidden layers by enabling the Transformer architecture to share fully connected layers and attention layers. This approach substantially reduces the number of parameters in Transformers of comparable size and markedly improves the training efficiency.

The pre-training process of the ALBERT model involves converting each token in the input statement into randomly initialized vector representations. Specifically, for the statement *S* = (*T*_1_, *T*_2_, *T*_3_, …, *T_t_*, …, *T_n_*), where *T_t_* represents the *t*-th token of sentence *S*, with *t* ranging from 1 to *n*, a sequence of tokens is formed. By employing the MASK masking mechanism during pre-training, the model can effectively capture the contextual semantic information of each token within the sentence.

By employing advanced parameter reduction techniques and the Sentence Order Prediction (SOP) task, ALBERT significantly reduces the number of model parameters while achieving substantial performance improvements in natural language processing tasks. Targeted training of the ALBERT model on specialized vocabulary within the domain of intelligent production line fault diagnosis can further enhance the accuracy of word embeddings and improve the precision of entity relationship extraction.

#### 3.4.2. BiLSTM Model

The Bidirectional Long Short-Term Memory (BiLSTM) network is an advanced architecture of Recurrent Neural Networks (RNNs) that incorporates Long Short-Term Memory (LSTM) units. It leverages the advantages of the Long Short-Term Memory mechanism while enabling bidirectional data processing. BiLSTM is particularly adept at capturing long-term dependencies in sequential data, allowing it to process both historical and future information simultaneously. This capability makes it especially well suited for time-series analysis, text processing, and various sequence prediction tasks. The LSTM unit consists of three essential gate structures: the input gate, the forget gate, and the output gate. These gates work collaboratively to effectively regulate the flow of information, optimize the memory and forgetting mechanisms of the network, and significantly enhance the BiLSTM’s ability to process complex sequential data.

In the context of sequence data analysis, Bidirectional Long Short-Term Memory (BiLSTM) networks effectively decrease the number of parameters while preserving their capacity to handle intricate data structures. This efficiency is attributed to a weight-sharing mechanism that functions across both the forward and backward LSTM layers. This architecture enables neurons at each time step to synthesize information from both directions: forward (left to right) and backward (right to left). BiLSTM networks proficiently mitigate the shortcomings associated with conventional Recurrent Neural Networks (RNNs) in the processing of sequential data. This is achieved by leveraging information from both forward and backward directions, which significantly improves the model’s ability to capture long-term dependencies and address challenges related to long-distance dependencies through the synthesis of outputs from both forward and backward LSTM components. For instance, in the named entity recognition (NER) task, methods leveraging BiLSTM–Conditional Random Fields (CRFs) have achieved excellent recognition results across various corpora. The BiLSTM can capture bidirectional dependencies in sequence data due to its robust contextual dependence capability and outstanding performance in tasks that require a comprehensive understanding of the overall context, such as named entity recognition. For example, the Attention–BiLSTM method for named entity recognition utilizes the BiLSTM neural network to automatically extract latent features from the text. The incorporation of the attention mechanism addresses the challenge of long-distance dependencies and enhances performance by evaluating the significance of global text features within the context. Therefore, BiLSTM has been widely used in various sequence modeling tasks in the field of natural language processing, including language model construction, text classification, and speech recognition, with the reasons being improved clarity, vocabulary, and technical accuracy while maintaining the original meaning.

The bidirectional network architecture of BiLSTM overcomes the limitations associated with the unidirectional processing of traditional LSTM. This capability enables BiLSTM to simultaneously capture both forward and backward information, thoroughly analyze contextual relationships, and effectively extract latent information from extensive texts. As a result, BiLSTM is selected for feature extraction from intelligent production line fault data, leveraging its ability to capture long-term dependencies and enhance the accuracy of fault analysis.

Using the vector output from the ALBERT pre-trained model and the token vectors from the sentence as inputs to the BiLSTM, the model learns both the forward sequence (*h*_1_, …, *h_t_*, …, *h_n_*) and the backward sequence (*h_n_*, …, *h_t_*, …, *h*_1_). The training process for the output vector of the BiLSTM can be described using Equations (1)–(5):

(1) $$f_{t}=\sigma \left(W_{f}T_{t}+Q_{f}\vec{h_{t-1}}+b_{f}\right)$$

(2) $$i_{t}=\sigma \left(W_{i}T_{t}+Q_{i}\vec{h_{t-1}}+b_{i}\right)$$

(3) $$o_{t}=\sigma \left(W_{o}T_{t}+Q_{o}\vec{h_{t-1}}+b_{o}\right)$$

(4) $$c_{t}=f_{t}c_{t-1}+i_{t}\times \tanh\left(W_{c}T_{t}+Q_{c}\vec{h_{t-1}}+b_{c}\right)$$

(5) $$\vec{h_{t}}=o_{t}\times \tanh\left(c_{t}\right)$$

In the above formulas, *f_t_*, *i_t_*, and *o_t_* represent the activation vectors of the forget gate, input gate, and output gate, respectively. *c_t_* represents the cell state vector; *h_t_* represents the BiLSTM output of the forward sequence at time step *t*. *W*, *Q*, and *b* are the weight, matrix, and bias vector, respectively, and *σ* denotes the sigmoid activation function. Therefore, the output vector is obtained by vertically concatenating the hidden state vector of the forward sequence *h_t_* with the hidden state vector of the backward sequence *h_t_*.

#### 3.4.3. Attention Mechanism

After completing the entity extraction task, the next critical step is to establish the relationships between entities and their attributes, a process known as relation extraction. Accurately extracting these relationships from complex texts is essential for comprehending the content. The application of deep learning technology in relation extraction has significantly improved both the efficiency and accuracy of relation classification by automatically learning extraction rules and features, as well as processing multimodal data. Consequently, building upon the previously developed named entity recognition model, we further integrate an attention mechanism to enhance the performance of relation extraction.

By introducing an attention weight value t in conjunction with the BiLSTM layer, we can significantly enhance the accuracy of entity type recognition by focusing on critical components of the input data, such as specific feature sequences. For instance, the attention-based named entity recognition model can more effectively identify entities in the text by learning contextual information, thereby improving the recognition accuracy of named entities. The attention weight corresponds to the *t*-th token, and the method for calculating it is detailed in Equation (6). By incorporating the attention mechanism, the model can more precisely concentrate on key segments of the text during relation recognition, thus enhancing both the accuracy and efficiency of relation extraction tasks.

(6) $$T_{t}=\frac{\exp\left(p_{t}\right)}{\sum_{i=1}^{n}\exp\left(p_{j}\right)}$$

In the input processing of the subsequent layer of the model, a dual-rail parallel approach is employed. First, the variable ‘*p_t_*’, as defined in Equation (7), is utilized to perform the token classification task, which involves the joint representation of the output ‘*h_t_*’ and the word vector ‘*T_t_*’ from the LSTM layer, as illustrated in Figure 3. This method aligns with the advanced application of LSTM in complex tasks, particularly those that require parallel processing across multiple GPUs. The joint representation [*T_t_*; *h_t_*] seamlessly integrates the semantic and contextual information of the current token within the sequence, resulting in a comprehensive fusion of data. Next, the *T_t_* function serves as the activation function for the attention layer, generating the attention weight *A_t_*, as shown in Equation (8). This enables the model to concentrate on identifying features that are closely related to the target task while processing tokens. Finally, the token vector *T_t_* and the BiLSTM outputs, weighted by attention, are concatenated to form a vector [*T_t_*; *T_t_h_t_*], which encapsulates both the token’s inherent information and contextual details. This information is enhanced through the attention mechanism, providing richer and more accurate input features for subsequent relation extraction tasks.

(7) $$p_{t}=\tanh\left(W_{p}\left[T_{t};h_{t}\right]+b_{p}\right)$$

(8) $$A_{t}=\tanh\left(W_{A}\left[T_{t};T_{t}h_{t}\right]+b_{A}\right)$$

#### 3.4.4. CRF Layer

As a foundational model, Conditional Random Fields (CRFs) play a significant role in the field of natural language processing. When integrated with the Bidirectional Long Short-Term Memory (BiLSTM) model, the CRF layer enforces syntactic rules and clarifies label relationships, thereby greatly enhancing the model’s predictive validity and accuracy. The incorporation of the CRF layer refines the output of the label sequence, ensuring that the resulting sequence adheres to the constraints of a highly probable sequence.

At the top of the attention layer, the Conditional Random Field (CRF) strategy is employed to classify a given token *T_t_* and predict the corresponding entity type for each token. Within the CRF framework, the score *S*(*A_t_*) for each entity label can be calculated using Formulas (9) and (10).

(9) $$S\left(A_{t}\right)=\tanh\left(W_{e}A_{t}+b_{sa}\right)$$

(10) $$p\left(\mathrm{ta}g\left|t_{t}\right.\right)=soft\max\left(S\left(A_{t}\right)\right)$$

In the context of named entity recognition (NER), the end-to-end model architecture effectively combines the strong sequence modeling abilities of Bidirectional Long Short-Term Memory (BiLSTM) networks with the label sequence optimization capabilities of Conditional Random Fields (CRFs). The CRF layer, by taking into account the global context of the entire label sequence, proficiently assigns the most suitable label to each token, thereby substantially improving the accuracy of entity-level recognition. This integration is particularly evident in the ALBERT–BiLSTM–CRF model, which demonstrates a significant enhancement in the performance of NER tasks.

#### 3.4.5. Joint Entity Relation Extraction

The input to the Conditional Random Field (CRF) layer for the subsequent layer consists of two components: the output state *h_t_* of the BiLSTM layer and the learned label embedding *l_t_*. The two components are concatenated to form a comprehensive feature vector *u_t_* = [*h_t_*; *l_t_*]. In the context of entity relation extraction, the CRF layer is crucial for identifying the most probable head entity vector along with its corresponding relation label from a sequence of tokens and a set of relation labels.

In the context of entity relation extraction, the score *S*(*u_j_*, *u_t_*, *u_l_*) between an entity token *T_t_* and an entity *T_j_*, given a specific relation label *r_t_*, is defined according to Equation (11). With the relation label *r_t_* specified, the probability of selecting the head vector token *T_j_* is calculated using Formula (12).

(11) $$S\left(u_{j},u_{t},u_{l}\right)=\tanh\left(W_{j}u_{t}+Q_{j}u_{t}+b_{j,t}\right)$$

(12) $$p\left(head=T_{j},label=r_{t}\left|T_{t}\right.\right)=soft\max\left(S\left(u_{j},u_{t},u_{l}\right)\right)$$

#### 3.4.6. Fault Diagnosis Knowledge Fusion of Intelligent Production Line

In the context of intelligent production line fault diagnosis, the construction of the data layer emphasizes two pivotal aspects: the extraction of diagnostic knowledge and its subsequent integration. Guided by the specifications of the ontology layer, this study employs an ALBERT–BiLSTM–Attention–CRF model, which incorporates attention mechanisms to enhance named entity recognition and relation extraction, thereby organizing the extracted information into structured triple data. However, fault diagnosis knowledge for intelligent production lines originates from a wide variety of sources. Due to the diverse nature of these data sources, the resultant knowledge often comprises ambiguous and fragmented elements, making it challenging to identify entities with similar connotations and elucidate their inter-relational connections. To address these issues, this paper employs information fusion technology to associate entities from various data sources that share the same semantics under a unified entity identity. This approach achieves coreference resolution and semantic alignment across diverse entity semantics, including those that share the same name, possess multiple names, or use abbreviations. This process ensures that the quality of knowledge meets usage standards, providing accurate and consistent structured knowledge for fault diagnosis in intelligent production lines.

## 4. Experiments and Results

### 4.1. Analyzing and Sourcing Multi-Source Fault Data

The fault knowledge graph serves as a sophisticated tool for knowledge management, utilizing a graph-based structure to represent entities associated with faults and their complex inter-relations [37]. This graph effectively consolidates various entities, including failure components, modes, symptoms, causes, and maintenance strategies, thereby clarifying their interactions through semantic links that illustrate causative relationships and dependencies among components. The amalgamation of diverse data sources, including equipment logs, historical fault records, maintenance manuals, and expert insights, in conjunction with data acquisition and real-time monitoring technologies, forms the basis for a robust knowledge base that supports fault diagnosis and maintenance management systems within automated production lines. This knowledge base not only encapsulates critical information regarding faults but also employs advanced analytical methodologies to assess the probability of occurrences, delineate the extent of impacts, and identify potential root causes. As failure cases are identified and resolved, the knowledge graph is continuously updated to ensure it reflects the most recent experiences in failure management and technological advancements.

The significance of intelligent production line fault data is highlighted by its extensive scale, diverse origins, complex structural characteristics, and valuable professional insights. The increasing complexity of industrial systems presents considerable challenges in managing and analyzing fault data, as evidenced by the difficulties associated with predictive maintenance and the need for advanced data analysis techniques. Within the realm of intelligent production line fault management, data knowledge is systematically categorized into three primary areas—knowledge related to the structure of production line equipment, maintenance practices, and fault analysis—with comprehensive details provided in Table 1. A comprehensive understanding of the configuration of production line equipment is crucial, requiring an acquaintance with its core principles, operational protocols, and procedures. These elements collectively form a robust theoretical framework that supports effective daily fault diagnosis and maintenance practices. The maintenance of production line equipment involves a diverse array of activities, including the monitoring of equipment status and the diagnosis and analysis of faults. This process not only aids in the development of practical maintenance experience, which is vital for fault identification and decision making, but also enhances the overall maintenance capability. Moreover, the analysis of production line faults synthesizes expert knowledge with valuable insights gained from historical fault cases, thereby establishing a thorough and resilient knowledge system for maintenance personnel in the field.

Table 1 clearly demonstrates that in the field of smart manufacturing, fault data predominantly exist in unstructured text format across production lines. To establish a fault knowledge graph, it is essential to convert these unstructured data into a structured triplet format. In this study, the key entities selected for constructing the knowledge graphs include the fault subject, content, cause, detection method, and corrective measures, while other relevant information serves as attributes. The subsequent section uses the example of an abnormal high-temperature fault in an industrial robot joint motor to illustrate the processing and representation of the original unstructured text data.

The primary concern regarding this issue is the joint motor of the industrial robot, which is notably characterized by an abnormal heating problem.

During the operation of industrial robots, the joint motors may reach temperatures that exceed the recommended operating range. This can result in performance issues and potential damage.

The causes of the failure are analyzed as follows: (1) Deviation of the power supply voltage from the rated standard can result in abnormal heating of the joint motor in an industrial robot, even during rated load operation. This phenomenon is primarily attributed to the temperature sensitivity characteristics of electrical insulation materials and permanent magnets. A voltage lower than the rated value increases motor losses, which, in turn, raises the temperature. (2) Frequent starts or excessive rotation cycles of the industrial robot’s joint motor can lead to significant heat accumulation. (3) High humidity levels in the working environment adversely affect heat dissipation efficiency.

Detection Method: (1) We measure the power supply voltage of the industrial robot to ensure it remains within the normal operating range. (2) To mitigate thermal energy accumulation, we enhance the robot’s trajectory planning by optimizing joint motor movements to minimize energy expenditure along both axes. (3) We implement strategies to reduce the ambient temperature and improve heat dissipation conditions.

To meet the training requirements of the deep learning model, the original text data undergo preprocessing. This process involves merging fault topics with their corresponding causes, descriptions, and detection methods into concise, self-contained sentences. Below is the preprocessed section:

The heating issue in the industrial robot’s joint motor becomes evident during operation, as it surpasses the normal temperature range.

The overheating issue in industrial robot joint motors is attributed to low power supply voltage and excessively high operating temperatures when functioning under rated load.

Overheating malfunctions in industrial robot joint motors are caused by excessive humidity in the working environment, which hinders effective heat dissipation.

The preprocessing technique effectively transforms raw text data into a structured format, facilitating seamless processing by deep learning models. This enhancement significantly improves fault diagnosis and predictive capabilities within intelligent production line systems.

### 4.2. Fault Data Labeling

Precise data labeling is essential for the successful construction of knowledge graphs and the effectiveness of deep learning applications, significantly influencing fault diagnosis and prediction. In the development of equipment fault knowledge graphs, the meticulous process of data annotation is indispensable [38]. It not only facilitates the extraction of critical information and the modeling of relationships but also significantly enhances applications such as fault diagnosis and maintenance. For example, the process of data annotation involves the identification and extraction of entities and their associated relationships, including component units, performance indicators, fault conditions, and detection tools sourced from diverse fault case documentation. This foundational work is essential for the subsequent development of fault knowledge graphs, the implementation of intelligent fault maintenance strategies, and the facilitation of real-time diagnostics within the high-end equipment manufacturing sector. Data annotation is fundamental to the advancement of machine learning, as it refines the quality of training data, enabling algorithms to more effectively comprehend the complex logic and subtle nuances within datasets. By leveraging precise annotations, the algorithm can uncover hidden patterns within the data, formulate highly accurate predictions, and efficiently execute the fault diagnosis task. Moreover, data annotation plays a pivotal role in enhancing algorithm accuracy by providing crucial background knowledge, thus empowering them to more accurately identify and understand fault characteristics.

At the ontology level, the BIOES (Begin, Inside, Outside, End, Single) format is employed to annotate entities within the original corpus, ensuring both consistency and accuracy in the annotation process [39]. This method enhances clarity in defining entity boundaries and hierarchies, thereby facilitating subsequent knowledge extraction and model training [40]. Table 2 presents a portion of the annotated data, illustrating the specific application of data annotation in the construction of a fault diagnosis knowledge graph. The sentence in Table 2, “工业机器人夹爪握力不足的排除方法是增加供气压力”, means “The elimination method of insufficient grip strength of industrial robot gripper is to increase the air supply pressure”.

### 4.3. Datasets

The smart production line fault dataset used in this study consists of 3255 fault record texts and 3125 fault analysis texts. To ensure the scientific rigor of model training and evaluation, the dataset was randomly divided into training, validation, and test sets in a standard ratio of 6:2:2. This division allocated 1953 fault record texts and 1875 fault analysis texts to the training set for training the named entity recognition model and the relation extraction model.

In the model training phase, the ALBERT–BiLSTM–CRF architecture is employed to train the named entity recognition model, while the ALBERT–BiLSTM–Attention architecture is utilized for training the relation extraction model. The partitioning strategy is crucial for ensuring that the model maintains its generalization capability and performance across diverse datasets, thereby establishing a robust foundation for subsequent optimization and validation.

### 4.4. Experimental Environment

In the training process of the knowledge extraction model, parameters are adjusted to enhance the model’s performance while effectively preventing overfitting. These parameters are configured to ensure optimal processing efficiency and memory management, with sentences limited to a maximum length of 128 tokens and a batch size set to 64. This configuration strikes a balance between memory usage and training efficiency. To further improve the model’s generalization ability, dropout is applied to both the input and output of the LSTM layer, with a ratio set at 0.2.

In each module of the model, the learning rates for the ALBERT, LSTM, and CRF layers are set to 0.0002, 0.001, and 0.001, respectively. This configuration enables differentiated control over the gradient update speeds of the various layers. The number of training iterations, or epochs, is set at 60. This setting facilitates rapid model convergence by ensuring an appropriate iteration frequency, thereby minimizing the risk of overfitting. The details of the software and hardware resources, as well as the environmental configuration required for this experiment, are provided in Table 3.

### 4.5. Evaluation of Knowledge Extraction Models

The evaluation index is essential for assessing the effectiveness of fault diagnosis models in intelligent production lines. This study employs a widely recognized deep learning evaluation framework to thoroughly evaluate the accuracy of extraction results, in accordance with established standards in fault diagnosis. Specifically, this study utilizes precision, recall, and the *F*_1_ score as the primary evaluation metrics, which offer a comprehensive assessment of the model’s predictive accuracy and reliability. The calculation formulas for each evaluation index are as follows (13)–(15):

(13) $$P=\frac{TP}{TP+FP}\times 100\%$$

(14) $$R=\frac{TP}{TP+FN}\times 100\%$$

(15) $$F_{1}=2\frac{P\times R}{P+R}\times 100\%$$

Here, precision (*P*) is defined as the proportion of accurately identified defects relative to the total number of detected defects, indicating the algorithm’s accuracy. Recall (*R*) is defined as the ratio of correctly identified defects to the total number of actual defects, serving as a measure of the algorithm’s completeness. *TP* represents True Positive, *FP* signifies False Positive, and *FN* denotes False Negative. These evaluation metrics are essential for quantifying and analyzing the model’s performance, thereby ensuring its effectiveness and practical application in intelligent production line fault diagnosis.

### 4.6. Results Analysis

In order to assess the efficacy of the ALBERT–BiLSTM–CRF model for entity recognition in fault texts associated with intelligent production systems, a series of comparative experiments were performed. These experiments aimed to evaluate the model’s performance in relation to established benchmarks within the domain of named entity recognition. This comparative study focused on models such as BiLSTM–CRF, which combines BiLSTM for feature extraction with CRF for decoding, thereby ensuring that the model accounts for label correlations within the sequence. Throughout the experiment, all models utilized the same training and test sets and were executed in a standardized experimental environment to ensure the comparability and fairness of the results.

**Figure 5:**
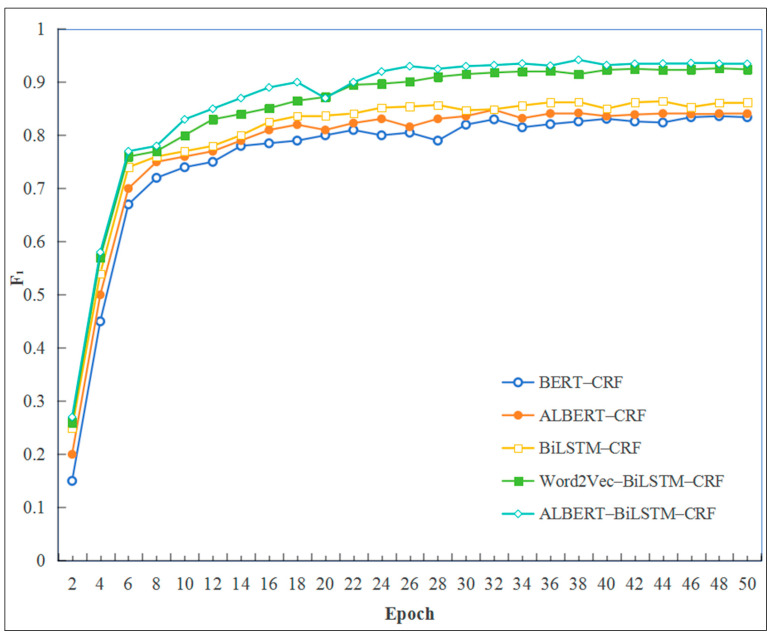
Comparison chart of *F*_1_ scores for entity extraction algorithms.

Following 40 to 50 iterations, stabilization is observed across all models, with the ALBERT–BiLSTM–CRF model exhibiting lower loss values during training. This outcome underscores the model’s superior performance compared to the other models under comparison. The ALBERT–BiLSTM–CRF model demonstrates robust performance on key evaluation metrics, achieving an average precision of 92.57%, a recall of 94.35%, and an *F*_1_ score of 93.45%. The observed performance indicates that the model is highly effective. Precision assesses the accuracy of positive predictions, recall measures the model’s ability to detect all positive instances, and the *F*_1_ score offers a comprehensive metric that combines both precision and recall. A detailed comparison of the experimental results is presented in Table 4 and Figure 5, highlighting the enhanced capability of the ALBERT–BiLSTM–CRF model to accurately identify entities within fault text data relevant to intelligent production lines.

Compared to the BiLSTM–CRF model, the ALBERT–BiLSTM–CRF model demonstrates a 7.3% improvement in the *F*_1_ score. This notable enhancement is primarily due to the ALBERT model’s capacity to effectively capture dynamic character features, which significantly enhances the model’s contextual understanding and improves the efficiency of extracting complex features from text.

In comparison to Word2Vec, the ALBERT–BiLSTM–CRF model enhances the *F*_1_ score by 1.05%. The fundamental difference between the two models lies in their respective embedding methodologies: Word2Vec employs a static approach, which struggles with polysemous words, whereas ALBERT utilizes a dynamic, character-based technique that offers a more effective solution to these lexical ambiguities.

As a pre-trained neural network model, ALBERT can achieve commendable training results even with limited text data. In the ALBERT–CRF model, the accuracy reaches 83.52%. Furthermore, the ALBERT–BiLSTM–CRF model, which integrates a Bidirectional Long Short-Term Memory (BiLSTM) network, demonstrates superior performance in the Chinese named entity recognition task, achieving an *F*_1_ score of 93.45%. Consequently, the ALBERT–BiLSTM–CRF named entity recognition model outperforms the comparative models in the experiment.

**Figure 6:**
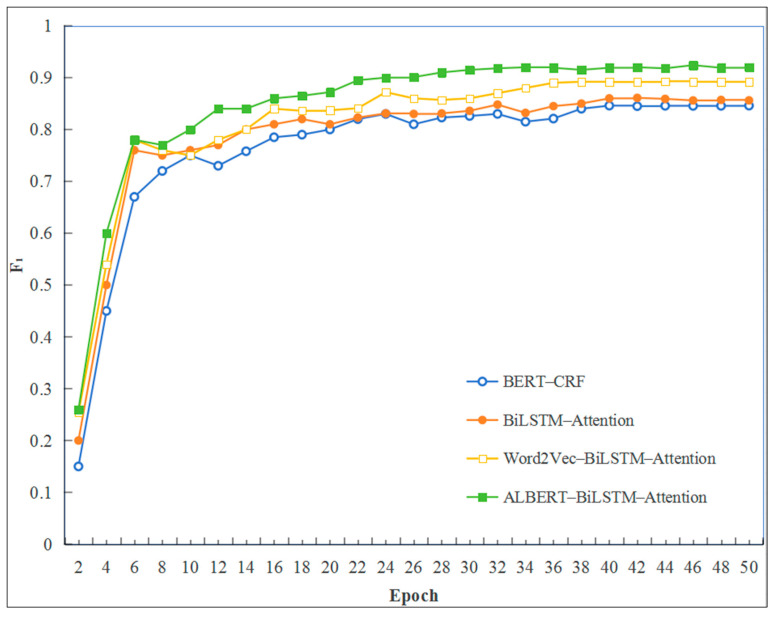
Relationship extraction model training curves.

To evaluate the efficacy of the ALBERT–BiLSTM–Attention relation extraction model proposed in this research, a comprehensive comparative experiment was conducted. This experiment effectively employs transformation testing to assess various relation extraction models. Within the scope of this experiment, the proposed model is compared with the conventional BiLSTM–Attention model and the BiLSTM–CRF model. The findings indicate that the ALBERT–BiLSTM model offers significant advantages over the alternative models, as supported by the detailed experimental results presented in Table 5 and Figure 6.

The findings presented in Table 5 and Figure 6 indicate that the ALBERT–BiLSTM–Attention model achieves a higher level of accuracy, supporting previous research that highlights the advantages of attention mechanisms in improving classification performance. Compared to the BiLSTM–CRF and BiLSTM–Attention models, the ALBERT–BiLSTM–Attention model shows a significant enhancement, with increases of 7.37% and 6.32% in the *F*_1_ score, respectively, due to the integration of the proposed attention mechanism.

## 5. Knowledge Storage and Application Case

### 5.1. Knowledge Storage and Visualization

**Figure 7:**
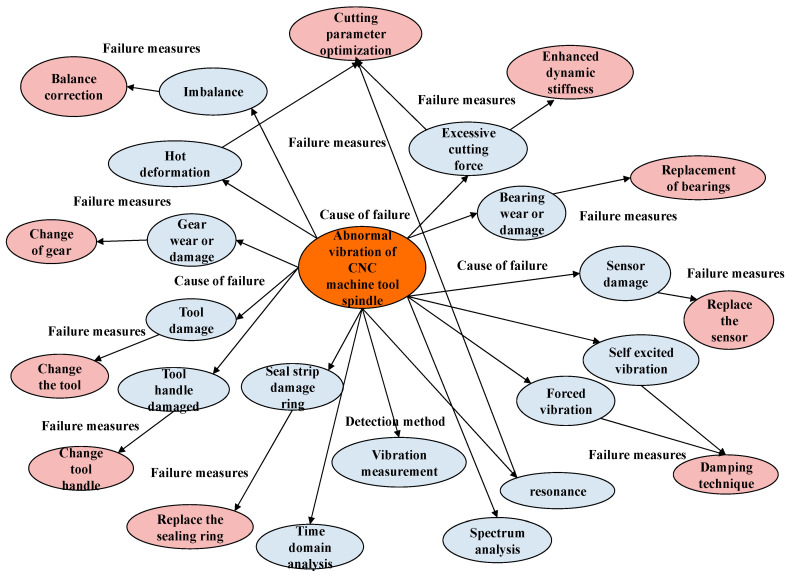
Fault knowledge diagram of NC machine tool spindle abnormal vibration.

By utilizing extensive fault data sourced from intelligent production lines and incorporating Neo4j graph database technology, we have successfully developed a sophisticated fault diagnosis knowledge graph specifically designed for intelligent production line systems [41]. Figure 7 illustrates a segment of the visualization content related to spindle vibration faults in CNC machine tools, as detailed in the knowledge map. This map offers a comprehensive overview of the potential causes and diagnostic techniques related to spindle vibration faults. It includes factors such as improper tool selection, inadequate cutting parameters, inappropriate speed adjustments, low precision in the engagement of gears and bearings within the spindle housing, the wear of guide rails, and unreliable clamping systems. Additionally, it addresses anomalies in spindle bearing temperatures, issues with spindle drive belts, problems with tool clamping, and mismatches between spindle speeds and feed rates.

In this visualization, each fault entity is represented as a node within the knowledge graph, while the inter-relations between these entities are depicted by edges that connect the head entity to the tail entity. The fault subject node is intricately linked with fault descriptions, detection methods, and maintenance procedures, creating a clear and comprehensible framework for fault diagnosis. The visualized knowledge graph significantly improves the accessibility and interpretability of information related to faults. Additionally, it serves as a valuable supplementary resource for maintenance personnel, facilitating more efficient fault analysis and decision-making processes.

### 5.2. Application Case of Fault Diagnosis and Auxiliary Analysis

**Figure 8:**
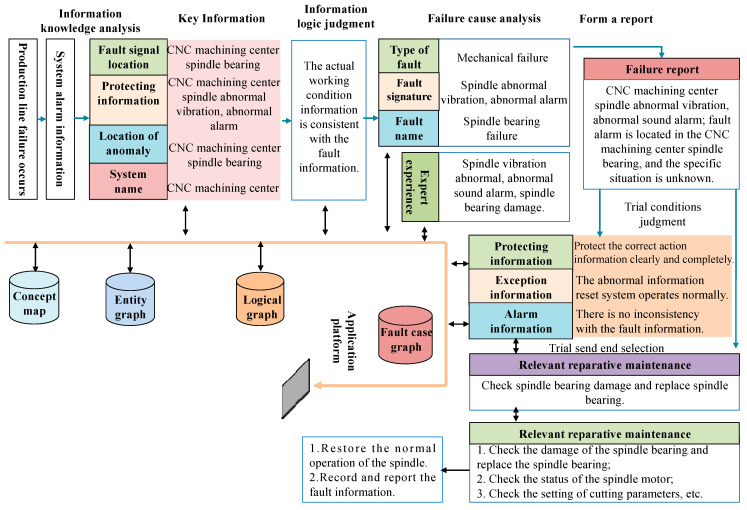
Application example of fault diagnosis of intelligent production line knowledge graph.

Taking the CNC machining center within an intelligent production line as an example, we demonstrate the practical efficacy of the fault diagnosis knowledge graph in identifying faults and facilitating auxiliary analysis. As demonstrated in Figure 8, the intelligent production line system effectively identifies faults, such as unusual vibrations and auditory alarms emitted by the spindle, by utilizing real-time visual and auditory data collected within the CNC environment. By employing advanced vibration analysis technology and high-precision sensors, the system utilizes the ALBERT–BiLSTM–Attention–CRF model to accurately detect both visible and audible signs of equipment malfunction.

In the context of CNC machine tools, the intelligent identification of fault modes is enhanced through a series of processes, including the collection of operational parameters, data preprocessing, feature extraction, and the application of support vector machine algorithms. Furthermore, traditional methods, including visual inspection and self-diagnosis, are commonly employed for fault detection in Computer Numerical Control (CNC) systems. The seamless integration of modern technology into equipment enhances predictive maintenance strategies, enabling earlier fault detection and facilitating the implementation of preventive measures. Concurrently, sensor data are utilized to continuously monitor production processes, ensuring that the preset thresholds for normal operation are not exceeded [42].

The system is designed to promptly trigger an alert mechanism upon identifying any discrepancies from the established parameters, thereby ensuring a swift response to potential anomalies. Moreover, the system integrates state-of-the-art sensors and controllers, meticulously monitoring and analyzing the machine’s operational status in real time, encompassing key indicators such as temperature, vibration, and pressure. Upon detecting a potential failure, the system automatically determines the specific failure mode [43]. Leveraging historical data and expert knowledge bases, deep learning algorithms have been developed to efficiently process complex data and predict the most probable failing component.

These algorithms automatically extract features and excel at managing high-dimensional, nonlinear fault data, thereby enhancing the accuracy of fault prediction. Upon confirmation of a fault, the system not only provides historical expertise in disposal and maintenance protocols but also identifies the specific components that require repair. This functionality facilitates a prompt response to faults and automatically recommends an appropriate treatment plan. The detailed procedure for fault disposal is outlined in the following section:

1. By integrating domain entity graphs, concept graphs, logic graphs, and fault case graphs, the comprehensive knowledge base effectively extracts critical data from alarm information in intelligent production lines, thereby enhancing the efficiency of fault diagnosis and response.
2. The fault signal is accurately identified, and a comprehensive analysis is conducted on protection information, abnormal locations, and system names. This process enables the determination of specific working condition details through logical reasoning, thereby clarifying the context surrounding the fault.
3. An in-depth causal analysis is conducted by integrating various fault types, their characteristics, and nomenclature, along with the expertise and knowledge of specialists. This approach facilitates the generation of comprehensive fault reports and provides decision-making support for subsequent fault management.
4. Careful analysis of the fault report; assessment of the status of the trial operation; review of the protection systems, alarms, and any abnormal information; and development of a feasible repair and maintenance strategy are carried out to ensure that the resolution of the fault is both safe and effective.
5. According to the analysis results, the fault disposal measures are developed and documented. Subsequently, these measures are uploaded and reported to facilitate the tracking and management of the fault resolution process.
6. Customized troubleshooting procedures that align with the proposed solution are leveraged to enable the rapid identification and resolution of issues.
7. After addressing the failure, the disposal process is documented and updated in the knowledge graph through knowledge extraction and storage. This critical step enhances subsequent fault-handling recommendations by continuously enriching and refining the knowledge graph, thereby improving the effectiveness of fault management.

### 5.3. Dynamic Update Mechanism for Fault Knowledge Graphs

In the fault diagnosis of intelligent production lines, a dynamic updating mechanism for the knowledge graph is essential for the real-time integration of new fault cases. In the event of new faults, the system utilizes sensor networks and data collection mechanisms to effectively monitor and gather relevant information. This includes, but is not limited to, equipment operating parameters, alarm signals, and maintenance records. This multi-source heterogeneous data are denoised and normalized before being transformed into a unified feature vector.

The system employs similarity matching technology to search for historical cases within the knowledge graph that align with the characteristics of a new fault, thereby providing preliminary diagnostic insights. Based on the matching results, the system integrates the new fault as an entity in the knowledge graph, creating or updating the relationships between entities. For instance, if the degree of similarity between the new fault and a historical case exceeds a specified threshold, a connection is established between the two instances. Simultaneously, relevant entity attributes, such as fault frequency and remediation strategies, are updated accordingly.

To enhance diagnostic capabilities, the system employs an incremental learning approach that integrates new fault cases into the model training process. By updating model parameters in real time, the knowledge graph can adapt to emerging fault patterns, thereby optimizing both the accuracy and recall rates of diagnostics. Furthermore, the system features a feedback mechanism that allows maintenance personnel to manually correct and supplement diagnostic results. This feedback is subsequently reintegrated into the knowledge graph, further enriching the knowledge system.

The implementation of this dynamic updating mechanism allows the knowledge graph to incorporate the latest fault information from the production line in real time, thereby providing comprehensive and precise contextual support for fault diagnosis. This approach not only enhances diagnostic efficiency for previously identified faults but also improves the ability to detect and address new fault types, resulting in a more accurate and effective intelligent fault diagnosis process.

## 6. Discussion

This research offers valuable insights into the development and application of a knowledge graph for fault diagnosis in intelligent production line equipment, with a particular emphasis on extracting information from knowledge graphs related to manufacturing faults. By identifying key entities and their inter-relations within fault case documentation, we establish a robust foundation for the subsequent construction of a fault knowledge graph and the implementation of intelligent fault diagnosis systems. This discussion explores the objectives and implications of the research, acknowledges its limitations, and outlines potential avenues for future investigation.

The study introduces an innovative methodology for diagnosing faults in intelligent production lines through the utilization of knowledge graphs. It successfully constructs a fault knowledge graph by integrating top–down pattern design with bottom–up data layer techniques. This achievement provides a novel approach to the systematic organization, efficient storage, accurate representation, and extensive application of fault-related knowledge within intelligent production lines. The proposed method effectively identifies fault patterns and their underlying causes, offering substantial data support and a decision-making framework for maintenance personnel. This underscores the practical significance of knowledge graphs in enhancing the operational efficiency and safety of intelligent production lines.

We acknowledge certain limitations inherent in this study, particularly concerning the ALBERT–BiLSTM–Attention–CRF model. While it demonstrates commendable performance, the associated computational costs are substantial. In resource-limited settings, future research may explore strategies for parameter sharing among modules, such as implementing parameter sharing between the BiLSTM and Attention components to alleviate computational demands. Additionally, we will consider employing matrix decomposition techniques on the weight matrix of the BiLSTM or the projection matrix of the attention mechanism to reduce the parameter scale and mitigate computational complexity.

Moreover, although the dataset utilized is comprehensive for the objectives of this research, the diversity of automated production line equipment and the complexity of defect scenarios suggest that additional data may be required to encompass all types and defect situations. Future efforts could concentrate on expanding the dataset to include a wider variety of equipment and defect classifications.

Despite the successful establishment of a knowledge graph for fault diagnosis in intelligent production lines, there remains significant potential for further enhancement and optimization. Future research endeavors will concentrate on the comprehensive optimization of model architectures and parameters, utilizing case studies that incorporate knowledge graphs for fault diagnosis. Additionally, there will be an exploration of various algorithms designed to enhance the optimization and evaluation processes. The objective is to improve the accuracy and completeness of the graph. Furthermore, by integrating advanced fault diagnosis technologies, such as multi-source data fusion techniques and deep learning algorithms, the model’s adaptability to a diverse range of faults in intelligent production lines will be significantly enhanced.

## 7. Conclusions

To improve the management and application of fault knowledge in intelligent production lines, thereby increasing the efficiency of fault diagnosis and ensuring the stable and reliable operation of these systems, a novel method for fault diagnosis driven by knowledge graphs has been introduced. This method involves the development of a fault knowledge graph specifically tailored for intelligent production lines. The key findings are as follows:

By leveraging knowledge graph technology, the integration of multi-source heterogeneous data in intelligent production lines has been accomplished, leading to the development of a fault diagnosis knowledge system that encompasses rich semantics and logical relationships. This system not only incorporates equipment information, fault manifestations, cause analysis, and solutions for the production line but also offers extensive knowledge support for fault diagnosis through semantic associations and logical reasoning capabilities.

A deep learning model based on the ALBERT–BiLSTM–CRF architecture has been developed, incorporating an attention mechanism to achieve precise extraction of key information from fault texts. This model utilizes the ALBERT layer for word embedding, the BiLSTM layer to process sequential data and generate scores for each word across various labels, and the CRF layer to ensure the validity of the final predictions through constraints. The model effectively identifies entities and relationships in fault texts, including equipment components, fault phenomena, cause analysis, and handling measures, thereby providing rich semantic information for fault diagnosis.

Finally, by developing a visualization platform for the knowledge graph associated with intelligent production line fault diagnosis and validating it through practical application cases, this platform offers maintenance personnel an intuitive tool for fault diagnosis and decision support. This not only allows maintenance personnel to quickly comprehend and utilize fault diagnosis knowledge but also improves the efficiency and accuracy of fault resolution.

## 8. Patents

In the course of this research, two Chinese invention patents relevant to this paper have been filed.

1. Yanjun Chen, Min Zhou, et al. Real-time monitoring system of automotive wheel production line based on digital twin. CN202411066233.1
2. Yanjun Chen, Min Zhou, et al. Construction method and system of intelligent manufacturing production line of automobile wheel based on digital twin. CN202411512597.8

## Figures and Tables

**Table 1:** Multi-source data management in intelligent production line systems.

Fault Data Type	Fault Data Structure	Selecting Content
Knowledge of production line equipment structure	unstructured	Equipment name, management guideline, operating procedure, maintenance record, scheduling protocol, operation date, etc.
Production line equipment maintenance knowledge	unstructured	Equipment name, fault type, fault condition, fault cause, disposal process, operation date, etc.
Knowledge of production line fault analysis	unstructured	Equipment name, expert experience, failure type, failure case, maintenance record, failure record, etc.

**Table 2:** Results of partial data annotation based on BIOES format.

Primitive Character	Labeling Results	Primitive Character	Labeling Results	Primitive Character	Labeling Results
工	B-object	力	I-wrong	是	O
业	I-object	不	I-wrong	增	B-method
机	I-object	足	E-wrong	加	I-method
器	I-object	的	O	供	I-method
人	I-object	排	O	气	I-method
夹	I-object	除	O	压	I-method
瓜	E-object	方	O	力	E-method
握	B-wrong	法	O		

**Table 3:** Experimental environment (hardware and software configuration table).

Serial Number	Configuration	Versions
1	System environment	Ubuntu18.04
2	Deep learning training framework	PyTorch1.8.1
3	Programming language	Python3.8
4	CPU	Intel(R)Core(TM)i5-12500 CPU @ 3.20 GHz 3.19 GHz
5	GPU	RTX 3090(24 GB)

**Table 4:** Comparison of experimental results of named entity recognition models (unit %).

Model	Accuracy (*P*)	Recall (*R*)	*F*_1_
BERT–CRF	81.32	83.43	82.36
ALBERT–CRF	83.52	84.63	84.07
BiLSTM–CRF	86.75	85.56	86.15
Word2Vec–BiLSTM–CRF	91.28	93.54	92.40
ALBERT–BiLSTM–CRF	92.57	94.35	93.45

**Table 5:** Comparison of experimental results of relation extraction model (unit %).

Model	Accuracy (*P*)	Recall (*R*)	*F*_1_
BiLSTM–CRF	85.47	83.64	84.55
BiLSTM–Attention	85.14	86.87	85.60
Word2Vec–BiLSTM–Attention	89.56	88.79	89.17
ALBERT–BiLSTM–Attention	92.58	91.26	91.92

## Data Availability

The original contributions presented in this study are included in the article. Further inquiries can be directed to the corresponding author.
